# A Green Approach for the Biosynthesis of Gold Nanoparticles Using *Cuminum cyminum L.* Seed and Its Application for Pain Management in Rats

**DOI:** 10.52547/ibj.26.3.219

**Published:** 2022-03-14

**Authors:** Sahar Golabi, Maryam Adelipour, Asma Mohammadi, Kosar Omidian, Ali Rastqar, Mahshid Naghashpour

**Affiliations:** 1 Department of Medical Physiology, School of Medicine, Abadan University of Medical Sciences, Abadan, Iran;; 2 Department of Biochemistry, School of Medical Sciences, Ahvaz Jundishapur University of Medical Sciences, Ahvaz, Iran;; 3 Department of Biochemistry, School of Medicine, Abadan University of Medical Sciences, Abadan, Iran;; 4 Department of Pharmacy and Nutrition, University of Saskatchewan, SK, Canada;; 5 Department of Psychiatry and Neuroscience, University of Medicine, Université Laval, Quebec, QC, Canada;; 6 Department of Nutrition, School of Medicine, Abadan University of Medical Sciences, Abadan, Iran

**Keywords:** Interleukin-6, Pain, Cuminum cyminum L., Gold Nanoparticles, Green Synthesis

## Abstract

**Background:**

This study investigated the antinociceptive effect of cumin and its biosynthesized AuNPs.

**Methods:**

Cumin extract (E) and cumin-AuNPs (GN) were prepared and administered intraperitoneally at the concentrations of 200, 500, and 1000 mg/ml to 27 male rats. UV–Vis spectroscopy and AFM were applied for AuNPs synthesis confirmation. The nociceptive behavior was assessed, and IL-6 serum levels were measured.

**Results:**

Cumin-AuNPs showed a peak absorption of 515 nm, and a size of about 40 nm. Three different concentrations of extract had no significant effect on acute and chronic nociceptive behavior. GN + E200 (46.00 ± 10.59) showed a significant acute anti-nociceptive effect compared to the control (98.66 ± 4.91; *p *= 0.029) and SS300 (98.33 ± 20.30; *p *= 0.029) groups. Also, GN + E500 (42.00 ± 11.84) significantly reduced acute nociceptive behavior compared to the control (98.66 ± 4.91; *p *= 0.019), SS300 (98.33 ± 20.30; *p *= 0.020), and GN + E1000 (91.00 ± 26.00; *p *= 0.040) groups. IL-6 serum levels reduced significantly in GN + E500 (24.65 ± 10.38; *p *= 0.002) and SS300 (33.08 ± 1.68; *p *= 0.039) compared to the controls (46.24 ± 3.02). Chronic nociceptive behavior was significantly lower in the SS300 (255.33 ± 26.30) compared to E200 (477.00 ± 47.29; *p *= 0.021), E500 (496.25 ± 46.29; *p *= 0.013), and GN + E500 (437.00 ± 118.03; *p *= 0.032) groups.

**Conclusion:**

Our findings suggest the potential effects of cumin-AuNPs on formalin-induced nociceptive behavior, which is independent of IL-6serum levels.

## INTRODUCTION

Pain is an indicator of active disease-promoting process during neurological disorders. The etiology of neurological diseases varied widely, including central nervous system traumatic injury, neurodegeneration, and neuroinflammation^[1]^. Cytokines and chemokines act as main inflammatory factors in pain caused by neuroinflammation^[2]^. Formalin as the most common in pain inducer^[3]^ is being used for the evaluation of the probable antinociceptive and analgesic effects of natural compounds and is sensitive to various classes of analgesic medications^[4]^. The formalin test is predominantly utilized to measure nociception in rats and mice. Classically, the formalin test includes two well-identified phases of spontaneous pain behaviors. This test is a suitable model to investigate the transition from acute to chronic pain^[6]^. Response to formalin shows an early and a late phase. The early phase seems to be caused predominantly by C-fiber activation due to the peripheral stimulus, while the late phase appears to depend on the combination of an inflammatory reaction in the peripheral tissue and functional changes in the dorsal horn of the spinal cord. These alterations are possibly initiated by the C-fiber barrage during the early phase^[5]^.

Nowadays, NSAIDs are widely used to reduce inflammation and relieve pain. Due to side effects induced by NSAIDs, the development of natural anti-nociceptive agents has been under focus^[7]^. SS, as an NSAID, irrevocably acetylates COX-1 and -2, inhibiting prostaglandin synthesis and related inflammation^[8]^.

Medicinal products from plant origin have shown therapeutic effects on many inflammatory situations with fewer adverse events^[7]^. Raw extracts of various types of medicinal plants and their isolated compounds have displayed antinociceptive and analgesic properties in several *in vivo* and *in vitro* studies^[9]^. *C. cyminum *L. (cumin) is a small herbaceous annual plant^[10]^. The available and usable parts of this plant are leaves, fruits, and seeds. Fruits of the cumin plant have been indicated to possess phenolic compounds^[11]^. Cumin is featured with antimicrobial, insecticidal, analgesic, and anti-inflammatory properties. Acetic acid induced writhing, hot plate, carrageenan-induced paw edema, and cotton-pellet granuloma models are commonly used to evaluate anti-inflammatory and pain-relieving effects of aqueous and alcoholic extracts of cumin^[10]^.

Various physical and chemical methods have been applied for the synthesis of NPs; however, some of these approaches have disadvantages such as using toxic solvents and unsafe products. Therefore, for the synthesis of metal NPs, it is necessary to develop eco-friendly methods^[12]^. Green nanotechnology is a rationale scientific method developed for over two decades to connect plant sciences and nano-technology and provides an inherently green approach to nanotechnology^[13]^. Plant-mediated synthesis of NPs has been received a wide attention owing to its intrinsic features, including rapidity, simplicity, eco-friendliness, and economics^[14]^. Nanomaterials have multiple applications in biology and medicine^[15-22]^, but the most important one is their use as anti-nociceptive agents^[23]^. Different green NPs with precise chemical composition, morphology, and size were synthesized by diverse methods, and their applications in many advanced technological areas have been discovered^[12]^.

Gold metal is universally used in combination with phytochemicals from several herbs including, green tea, cinnamon, clove, gooseberry, grape, mango, and turmeric^[12,24]^. Phytochemicals are indeed electron-rich antioxidants, therefore, the interaction of gold metallic precursors with phytochemically-harnessed electrons produce herb/phytochemicals-encapsulated, well-defined, AuNPs^[24]^. The large surface area of AuNPs embedded with atomically active nanosurface motif allow highly efficient encapsulation of phytochemicals ^[24]^. Recently, the application of AuNPs has been widely evaluated as a promising approach in the diagnosis and treatment of various medical situations^[25]^. An *in vitro* study has shown the anti-inflammatory and analgesic effects of biosynthesized (plant extracts-mediated) AuNPs through inhibiting the secretion of inflammatory mediators^[26]^. Moreover, preclinical *in vitro* and *in vivo* studies on the effect of AuNPs-based Nano Swarna Bhasma drug with a cocktail of phytochemicals showed an ability to reduce tumor volume in the treated group of breast tumor-bearing mice. Also, a pilot human clinical trial unequivocally established that the innovative green nanotechnology used for treating metastatic breast cancer patients exhibited dose-dependent efficacy in cancer cells death and confirmed that the Nano Swarna Bhasma drug is selectively toxic to tumor cells with minimal or no toxicity against the normal human cells^[24]^.

The neuroimmune system plays a precious role in the development of pain. Levels of pro-inflammatory cytokines (e.g. IL-6 and TNF-α) increase in the central nervous system, contributing to the pathophysiology of pain^[27]^. It has been shown that among healthy adults who experienced a sequence of psychophysical pain testing procedures, IL-6 elevates in the first hour after various pain induction procedures and remains high to one hour post experience^[28]^. Our recent investigation on green synthesized silver NPs led to introduce the antinociceptive properties of new AgNPs synthesized from cumin seeds and showed these features in formalin-induced pain^[29]^.

Given the increasing importance of herbal medicine in different fields of medical sciences such as pain study, diverse adverse effects of chemical treatments in pain management, the crucial role of nanotechnology in medicine, and the possible antinociceptive features of NPs, the present study was carried out to evaluate the antinociceptive effects of cumin seeds aqueous extract and biosynthesized AuNPs from cumin. We also assessed the possible role of IL-6 serum levels in an animal model of acute and chronic nociceptive behavior.

## MATERIALS AND METHODS

**Animals and study groups**

 This study was conducted on 27 male Wistar rats weighing 200-250 g. The rats were obtained from the Animal Breeding Facility at Ahvaz Jundishapur University of Medical Sciences (Khuzestan, Iran) and were kept in the Animal Housing Room of Abadan University of Medical Sciences (Khuzestan) for one week to adapt the laboratory environment. Animals were maintained in suitable moisture at 22 ± 2 °C on a 12:12 light-dark condition with free access to adequate water and food supplies and randomly assigned to nine experimental groups (n = 3 in each group)^[30,31]^ as follows: (1) the control group, intact rats receiving a single injection of 50 μl of 2% subcutaneous formalin into the right hind paw, which was examined by formalin test; (2) the vehicle group, receiving a single injection of intraperitoneal normal saline (mg/kg body weight); (3) three intervention groups receiving a single intraperitoneal injection of cumin extract (E) at the concentrations of 200 (E200), 500 (E500), and 1000 (E1000) mg/kg body weight ^[32]^; (4) three additional intervention groups receiving a single intraperitoneal injection of cumin-AuNPs (GN + E) in doses of 200 (GN + E200), 500 (GN + E500), and 1000 (GN + E1000) mg/kg body weight; (5) the positive control group (SS300) receiving a single intraperitoneal injection of SS in a dose of 300 mg/kg body weight. Groups 2-5 received the injection 30 minutes before the formalin test^[32]^.

**Preparation of** ***C. Cyminum*** **L.** **extract **

**Fig. 1:**
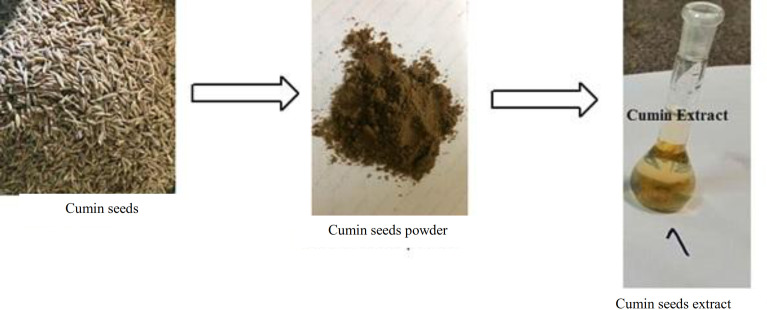
The scheme of the preparation of aqueous extract of cumin seeds.

Cumin seeds were purchased in July 2019 (Kerman, Iran). Seeds were washed to eliminate any contamination and placed under sunlight for a week to completely remove the moisture. For the preparation of extracts with different concentrations, an electrical grinder was used to crush five grams of dried seeds of cumin. The obtained powder was mixed with 50 mL of deionized water and kept on a rotatory shaker for 24 h. The mixture was boiled at 100 °C, filtered twice through a Whatman no.1 filter paper and centrifuged at 5000 ×g for 15 min. After collecting the supernatant, the solvent was evaporated at 40 °C by a rotary evaporator. The final volume of the extract was brought to desired concentrations (200, 500, or 1000 mg/mL) using saline. The extracts were sealed in an air-tight manner and preserved at 4 °C till further usage^[33-35]^. The scheme of the cumin seed extract preparation method is shown in Figure 1.

**Biosynthesis of AuNPs using cumin extract**

**Fig. 2:**
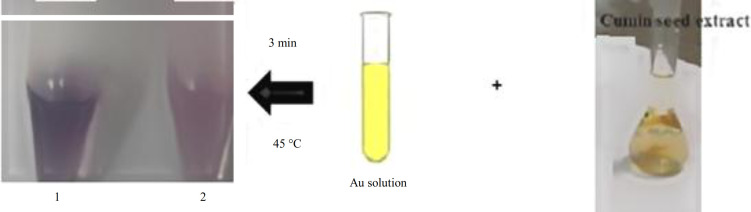
Production of cumin-AuNPs through green nanotechnology. Tubes number 1 and 2 show cumin-AuNPs solutions in doses of 500 and 200 mg/mL, respectively.

 In this study, an established biosynthesis technique was developed for the preparation of AuNPs using *C. cyminum *L. seed extract^[36]^. For this purpose, 2 mL of cumin extract with different concentrations were added to the ultrapure water in a test tube and slowly stirred in the water bath (at 40 °C). Then 1 mL of aqueous gold solution was added to the mixture. The change in the color of mixture from yellow to purple-red was the benchmark of AuNPs formation (reduction of Au^3+^ to Au^0^)^[13]^. The cumin-AuNPs synthesis method and different concentrations of biosynthesized AuNPs are shown in Figure 2. Two influential parameters, i.e. the temperature of the water bath and reaction time, were tested and optimized. Temperature ranges from 20 °C-60 °C of the water bath were investigated^[20]^. The effective formation time of AuNPs was studied at a range of 2-10 minutes. Color change in the biosynthesis of NPs indicated the completion of the reaction and NS formation.

Characterization of AuNPs

*UV–Vis spectrophotometry analysis of AuNPs*

The change in the color of the cumin extract-Au solution from yellow to purple-red indicated that the formation of AuNPs was completed. After the completion of the reaction, the scanning UV–Vis spectrophotometry for the synthesized cumin-AuNPs was carried out by recording a range between 400-700 nm.

*AFM of AuNPs*

The morphology and size of the biosynthesized AuNPs were determined by AFM. The AFM samples were prepared by spin coating the AuNPs solution into the glass slide. The thin film of a few drops of AuNPs were deposited on a silica glass plate in the darkness at room temperature. The deposited film glass plate was then scanned with the AFM. The prepared slides were air-dried and subjected to AFM analysis.

**Formalin test**

The formalin test was conducted based on a model developed by Tjolsen *et al.*^[5]^. All animals were weighed before the test and then placed in the formalin test cage for adaptation. Then 50 μl of 2% formalin was injected subcutaneously to the right hind paw of the rats. Next, the animals returned to the formalin test cage to count the number of flinching/shaking behavior at an interval of 1-min periods for a total observation duration of 60 min. The paw flinches/shakes behavior was assessed in an observer blind manner and considered as a nociceptive response in the animals. The acute and chronic phases of the formalin-induced nociceptive behavior were defined as the period immediately after the injection of formalin until 10 minutes and 20 to 60 minutes after formalin injection, respectively. There was a quiescent interphase of 10 minutes when the animals showed very little nociception behavior^[5]^.

**Measurement of serum IL-6**

After the behavioral tests, the animals were exposed to light ether anesthesia and euthanized according to the ethics of working with laboratory animals. Blood samples were taken from the heart to measure IL-6 serum levels. Blood samples were centrifuged, the serum separated and kept at -80 °C until testing. IL-6 serum levels were evaluated by rat standard ELISA kit (Zellbio, Germany) according to the manufacturer’s protocol.

**Statistical analysis**

The Kolmogorov-Smirnov and Shapiro-Wilk tests were used to determine the normal distribution of the parameters. The investigation of Skewness and kurtosis was also conducted using descriptive statistics. The data of the acute and chronic phases of the formalin test were analyzed separately using one-way ANOVA and Post Hoc Fisher’s LSD to detect which pairs of means are statistically different. Also, the comparison of the study groups in terms of IL-6 serum levels was analyzed using the Kruskal-Wallis test, followed by LSD Post Hoc tests. SPSS software version 21 was applied to analyze the data. Mean ± standard error of mean (SEM) was used to express the parametric data. Also, the results of the non-parametric test were expressed as the mean rank ± SEM. The significance level of difference was set at p < 0.05.

## RESULTS

**Optimization of gold ions reduction into AuNPs**

**Fig. 3:**
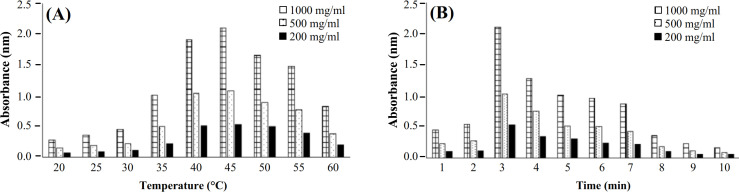
(A) The results of the temperature (ranges from 20 °C-60 °C) and (B) The results of the reaction time (ranges from 2 to 10 minutes) effect on the synthesis of different concentrations of cumin-AuNPs. The experiment was repeated at the intervals of five degrees and intervals of one minute.

Various temperatures of the water bath were investigated in the range of 20 °C-60 °C, which revealed the appropriate temperature of 45 °C for the cumin-AuNPs formation. The results of the temperature effect on cumin-AuNPs synthesis are shown in Figure 3A. Reaction time was investigated at time ranges from 2.0-10.0 minutes. The reaction time of three minutes at 45 °C was selected as the optimum time for the formation of cumin-AuNPs. The results of the reaction time effect on cumin-AuNPs synthesis are shown in Figure 3B.

**Cumin-AuNPs characteristics**

**Fig. 4:**
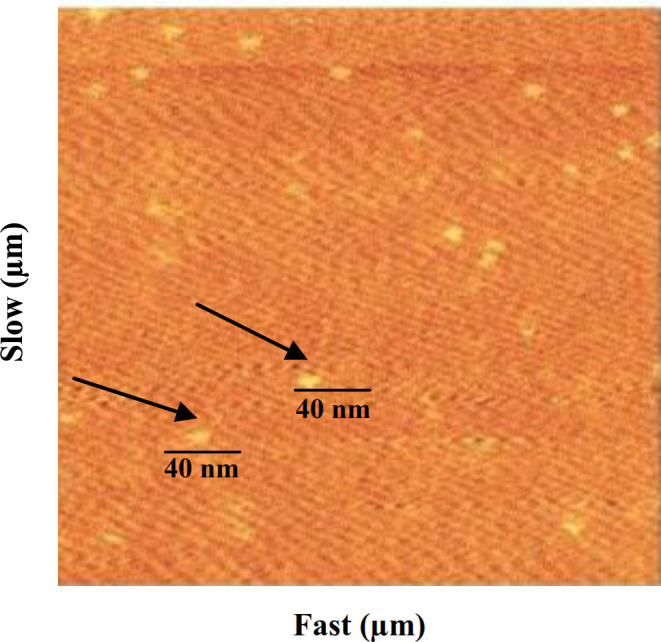
UV–Vis absorption spectrum of cumin-AuNPs in different concentrations. The synthesized AuNPs solution was detected by UV-Vis spectrophotometer at different wavelengths (400-700 nm).

**Fig. 5:**
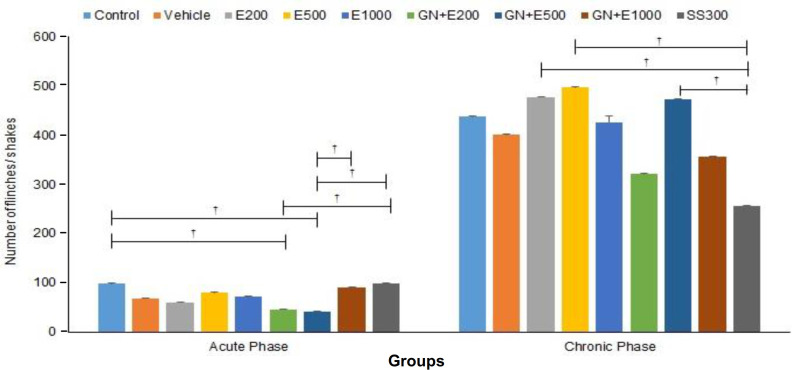
Characterization of AuNPs shown by AFM. The size of AuNPs in the sample (cumin-AuNPs 500 mg/ml) was about 40 nm. Microscope imaging speed is indicated as slow and fast in the Figure.

Cumin-AuNPs synthesis was confirmed using UV-Vis spectroscopy. The UV-Vis spectrophotometry was recorded in the range of 400–700 nm. AuNPs had a maximum wavelength of around 500-600 nm^[13]^. The spectra obtained after the completion of the reaction showed that the maximum absorption peak shifted to a wavelength of 515 nm. Figure 4 shows the Uv-Vis spectra recorded for cumin-AuNPs at different concentrations. The morphological features of cumin-AuNPs were evaluated by AFM. The AFM result of the cumin-AuNPs is presented in Figure 5. According to the obtained results, the size of cumin-AuNPs was about 40 nm.

**Effect of different concentrations of aqueous extract of cumin and cumin-AuNPs on acute nociceptive behavior**

**Fig. 6:**
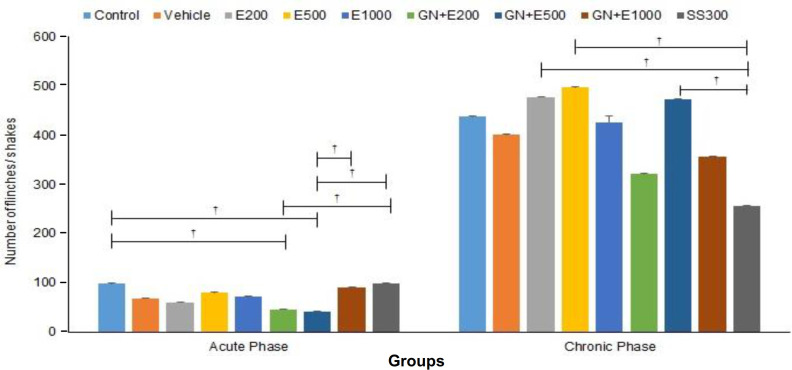
Effect of different concentrations of cumin aqueous extract (E200, E500, and E1000) and cumin-AuNPs (GN + E200, GN + E500, GN + E1000) on acute and chronic nociceptive behavior. (n=3 and ^†^*P* <0.05)

The formalin-induced nociceptive behavior of rats was assessed by counting the number of flinches/shakes of formalin-injected paws as 1 min passes throughout the recording time. The results of the observations during the early acute phase of formalin-induced nociceptive behavior are illustrated in Figure 6. The results revealed that acute nociceptive behavior reduced significantly in GN + E200 and GN + E500 (*p = *0.029 and *p = *0.019, respectively) compared to the control groups. Moreover, the rats in GN + E500 showed a significantly lower acute nociceptive behavior compared to the rats in GN + E1000 and SS300 groups (*p = *0.040 and *p = *0.020, respectively). Also, acute nociceptive behavior reduced significantly in GN + E200 compared to the SS300 group (*p = *0.029). However, acute nociceptive behavior showed no significant difference between the control and vehicle groups (*p *> 0.05). Comparison of acute analgesic effect between different doses of cumin extract and cumin-AuNPs showed no significant difference (*p *> 0.05).

**Effect of different concentrations of aqueous extract of cumin and cumin-AuNPs on chronic nociceptive behavior**

The results of the observations during the late tonic phase of formalin-induced nociceptive behavior are illustrated in Figure 6. The results showed that chronic nociceptive behavior decreased significantly in the SS300 compared to E200 (*p *= 0.021), E500 (*p *= 0.013), and GN + E500 (*p *= 0.032) groups. Also, the intraperitoneal injection of an effective dose of SS, 200, 500, and 1000 mg/ml of cumin extract, and 200, 500, and 1000 mg/ml of cumin-AuNPs indicated no significant effects on chronic nociceptive behavior (*p *> 0.05). Comparison of chronic analgesic effect between different doses of cumin extract and cumin-AuNPs showed no significant difference (*p *> 0.05).

**Effect of different concentrations of aqueous extract of cumin and cumin-AuNPs on IL-6 serum levels**

**Fig. 7:**
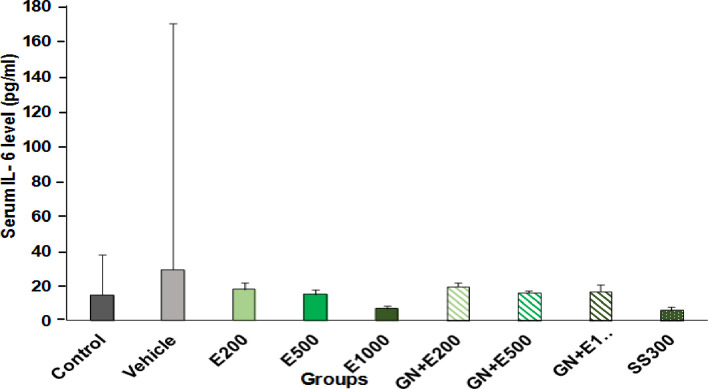
Comparison of the effect of different concentrations of cumin aqueous extract (E200, E500, and E1000) and cumin-AuNPs (GN + E200, GN + E500, GN + E1000) administration on IL-6 serum levels during formalin-induced pain. (n=3, *P*=0.054).

IL-6 serum levels were measured using ELISA kit in different groups. Figure 7 shows the comparison of mean rank and SEM of IL-6 serum levels between study groups analyzed by the Kruskal-Wallis test, followed by LSD Post Hoc tests. No significant difference was found between E200, E500, E1000, GN + E200, GN + E500, GN + E1000, and SS300 and the control groups (*p *= 0.054).

## DISCUSSION

The current study was conducted to investigate the possible antinociceptive activity of cumin seeds aqueous extract and the cumin-AuNPs in an established animal model of pain induction and evaluation. Also, the possible role of IL-6 in this process was investigated. To our knowledge, this is the first study demonstrating the antinociceptive potentials of the biosynthesized AuNPs using cumin seeds aqueous extract in the formalin model of pain. We found that the cumin-AuNPs exerted promising acute antinociceptive effects in the formalin model of pain. Meanwhile, our results indicated that biosynthesized AuNPs using cumin extract attenuated formalin-induced nociceptive behavior independent of IL-6 serum levels.

In the acute phase of the experiment, the administration of the cumin-AuNPs in doses of 200 and 500 mg alleviated the acute nociceptive behavior. Simultaneously, the administration of the different doses of cumin-AuNPs did not decrease IL-6 serum levels. It has been shown that the *C. cyminum* plant contains various phytochemicals such as γ-terpinene, α-pinene, linalool, and β-pinene, pinocarveol, cumin, and carotol, with powerful antioxidant characteristics and the ability to scavenge some free radicals. This antioxidant activity may exert an anti-inflammatory effect because of the mutual and important relationship between inflammation and oxidative stress^[37,38]^. However, in our study, the nociceptive effects observed with cumin-AuNPs administration were independent of IL-6 serum levels. It has been reported that AuNPs synthesized by the extract of *Panax ginseng* *Meyer *reduce the gene expression of inflammatory mediators such as IL-6 and TNF-α and subsequently decrease inflammation through inhibiting the activation of nuclear factor-κB downstream in macrophages^[39]^. AuNPs are characterized by non-toxic and high absorption features, which are mainly excreted in urine without any toxicity^[40]^. Moreover, phytochemicals in cumin are excellent coatings on AuNPs. By mixing cumin extract with sodium tetrachloroauratefor, they biosynthesized AuNPs that has nontoxic properties^[13]^. Taken together, the AuNPs may be valuable to suppress the production of proinflammatory mediators or activity in an animal model of inflammatory pain. In our study, it seems that the observed antinociceptive feature of AuNPs is carried out through other pathways independent of IL-6 serum levels, which may involve altering serum levels of other cytokines and chemokines, scavenging the reactive oxygen species radicals, decreasing lipopolysaccharide induced cytokine production, and modulating mitogen-activated protein kinase and phosphatidyl inositol 3-kinase pathways^[41]^. 

 In parallel with our study, a study on male Albino mice with acute and chronic inflammation induced by formalin indicated that cumin aqueous extract in doses of 200, 500, and 1000 mg/ml modulates acute and chronic pain^[42]^. Since the analgesic effect of cumin-AuNPs in a dose of 500 mg/ml was greater than the sodium salicylate, and because the administration of the cumin extract did not have any significant effect on the acute nociceptive response, it likely seems that the AuNPs boost the acute antinociceptive effect of cumin compared to the sodium salicylate. AuNPs may improve drug delivery via regulating the cell membrane penetration by surface ligand arrangement. Therefore, changes in the structure of the cell membrane could be a possible cause for increased cytoplasmic leakage in treatment with cumin-AuNPs^[25,43-45]^. This mechanism may explain the probable positive impact of cumin-AuNPs administration on the improvement of the acute antinociceptive feature. Similarly, a green nanotechnology investigation focused on the production of resveratrol-conjugated AuNPs indicated evidence of apoptotic events in human breast, pancreatic, and prostate cancer cell lines. It is necessary to explain that resveratrol, like many other plant-based phytochemicals in medicine, suffers from poor bioavailability once administered *in vivo*. This limitation is likely due to its susceptibility to rapid enzymatic degradation by the innate immune system of the body before it can exert its therapeutic influence^[46]^. Also, cumin may be codelivered using the AuNPs delivery device. Therefore, codelivery of AuNPs with cumin may have been various potential advantages, e.g. synergistic effects, suppressed drug resistance, and the ability to tune the relative dosage of cumin to the level of a single AuNPs carrier. Furthermore, the NPs carrying aptamers are able to target the delivery and uptake of their cargos to a subset of cells while simultaneously acting as fast releasing carriers for hydrophilic intercalating drugs^[47]^.

It can be postulated that in our study the AuNPs may improve the possible acute antinociceptive effect of cumin via a targeted delivery or codelivery of cumin extract. However, we did not examine the possible synergistic effect against the formalin pain because we did not give cumin-AuNPs and cumin extract or cumin-AuNPs and SS300 to the animals at the same time. To elucidate the mechanism of this synergistic nociceptive effect, elaborate experiments will be required. In general, the approach of using a combination of analgesics and AuNPs seems to be one of the best strategies for pain control and, therefore, therapeutic management in pain control. To our knowledge, there are no studies to evaluate the synergistic effects of AuNPs synthesized from cumin extract and analgesics. To accurately determine the effect of cumin-AuNPs on nociceptive behavior, we investigated the antinociceptive effects of different concentrations of cumin extract and the cumin-AuNPs. The results showed that in the early acute phase of formalin-induced nociception, the cumin-AuNPs in a dose of 500 mg reduced acute nociceptive behavior compared to the cumin-AuNPs in a dose of 1000 mg independent of IL-6 serum levels. This result revealed that in the early acute phase, the antinociceptive effect of different concentrations of the cumin-AuNPs does not follow a dose-dependent pattern. Previous studies have confirmed a complex of pro- and anti-inflammatory mediators involved in the pathophysiology of inflammation, leading to pain. It has also shown that various chemical agents in the plant extracts can affect the production and activity of different inflammatory mediators, including cytokines. Hence, they have different inhibitory and stimulating effects depending on many factors like their dosage or the method of extraction^[48]^. It has formerly demonstrated that varied doses of plant extracts may exert different influences on the inflammatory system^[32,49,50]^.

The administration of different doses of cumin aqueous extract and the cumin-AuNPs showed no significant chronic antinociceptive effect. A study proved that the cumin seeds aqueous extract had predominant acute anti-inﬂammatory activity, while the ethanol extracts of cumin seeds demonstrated primarily analgesic activity in the chronic phase of inflammation. This finding indicates that the analgesic action of the cumin extract in the chronic phase of inflammation might be due to the potentiation of opioid-like effect and reduction in the production and release of nitric oxide^[51]^. Also, in an experimental study, the essential oil of cumin revealed a significant and dose-dependent analgesic impact on the chronic but not acute model of inflammatory pain. The essential oil was devoid of anti-inflammatory activity^[52]^. The results of these studies indicate that different medicinal formulations of cumin may have variable effects on the acute and chronic phases of inflammation and pain^[51,52]^. It has also been described that there is a strong possibility that the inhibition of the COX enzyme by cumin may be the reason for its analgesic activities in the chronic phase of acetic acid-induced writhing test in Swiss Albino mice^[51]^.

Similar to the the study mentioned above, the NSAIDs (SS) administration in the present study significantly reduced the chronic inflammation compared to the cumin extract in the doses of 200 and 500 mg and the cumin-AuNPs in a dose of 500 mg. This evidence suggests that the chronic phase is sensitive to NSAIDs^[53]^. Following experimental studies, the late phase of the formalin test may depend on the combination of a nociceptive response in the peripheral tissue and functional changes in the dorsal horn of the spinal cord^[5]^. It seems that the acute analgesic effects of cumin-AuNPs are due to their effect on the inflammatory reactions of the paw tissue (local effect). Experimental studies conducted in the 1990s had indicated that serotonin, histamine, prostaglandins, and excitatory amino acids contribute to the late phase of formalin-induced inflammation^[54-57]^. Therefore, it is speculated that there is a possible interaction between the active constituents of cumin extract and mediators involved in peripheral inflammation. To support this hypothesis, Srivastava^[58]^ has reported that the ether extract of cumin inhibits eicosanoid synthesis along with an increase in the formation of lipoxygenase-derived products. Moreover, it has been described that myrcene, as one of the constituents of cumin essential oil, possesses a peripheral analgesic effect on the hyperalgesia induced by prostaglandins in the rat paw test. Thus, it can contribute to the analgesic effect of cumin*.* One of the limitations of the present study was the short duration of the study (one hour), which seemed insufficient for a comprehensive study of the effectiveness of the cumin extract and its biosynthesized AuNPs on chronic inflammation and nociception. Also, in this study, we did not apply the dynamic light scattering and the electrophoretic light scattering techniques to investigate the size and zeta potential of AuNPs, respectively, as well as the SEM and transmission electron microscopy to determine the morphology of AuNPs. Moreover, the identification of synthesized AuNPs should be confirmed by the X-ray diffraction test. Applying these techniques should be considered in future studies.

Our results suggested that the optimum temperature and reaction time for the biosynthesis of AuNPs using cumin seeds aqueous extract were 45 ºC and 3 minutes, respectively. Moreover, the biosynthesized AuNPs has significant antinociceptive activities in acute pain. Our findings support the potential therapeutic application of AuNPs for acute nociception. Given the little toxicity of cumin and anti-nociceptive mechanism of the AuNPs, the results of the present study can be applicable for cumin-based drug designing and targeting as well as in the food industry and also offer a probable solution for the prevention or treatment of acute inflammatory pain with minimal side-effects. However, broad-spectrum studies on specific cellular and molecular mechanisms of action, as well as controlled clinical trials to prove its efficacy in humans, are required to further assess the application of cumin and its biosynthesized gold nanoparticles as an anti-nociceptive agent.

## DECLARATIONS

### Acknowledgments

The authors would like to express their deepest gratitude to all staff and supervisors of the Abadan University of Medical Sciences (Khuzestan, Iran) for their contribution to this study. 

### Ethical statement

All experiments were conducted under the guidelines for animal care and use, by the U.S. National Institutes of health agency (pub: 80-23, revised in 1996). All testing approaches were proved by the Ethics Committee of the Abadan University of Medical Sciences (Ethical code: IR.ABADANUMS.REC.1396. 219).

### Data availability

The analyzed data sets generated during the study are available from the corresponding author on reasonable request.

### Author contributions

SG, MN, MA: conceptualization; SG, AM, and MA: methodology; SG, AM, MN, KO, and AR: investigation; SG and MN: writing original draft; SG, MN, KO, and AR: writing, review, and editing; SG: funding acquisition and supervision.

### Conflict of interest

None declared.

### Funding/support

This study was financially supported by the Abadan University of Medical Sciences (Grant NO. 96U-12).

## References

[1] Borsook D (2012). Neurological diseases and pain. Brain.

[2] Sommer C, Leinders M, Uceyler N (2018). Inflammation in the pathophysiology of neuropathic pain. Pain.

[3] Gregory NS, Harris AL, Robinson CR, Dougherty PM, Fuchs PN, Sluka KA (2013). An overview of animal models of pain: disease models and outcome measures. The journal of pain.

[4] Yoon MH, Bae HB, Choi JI (2005). Antinociception of intrathecal adenosine receptor subtype agonists in rat formalin test. Anesthesia and analgesia.

[5] Tjolsen A, Berge OG, Hunskaar S, Rosland JH, Hole K (1992). The formalin test: an evaluation of the method. Pain.

[6] Zhang L, Yin JB, Hu W, Zhao WJ, Fan QR, Qiu ZC, He MJ, Ding T, Sun Y, Kaye AD, Wang ER (2018). Analgesic effects of duloxetine on formalin-induced hyper-algesia and its underlying mechanisms in the CeA. Frontiers in pharmacology.

[7] Kumar S BB, Kuldeep S, Kalia AN (2013). Anti-inflammatory activity of herbal plants: A review. International journal of advances in pharmacy, biology and chemistry.

[8] Xu XM, Sansores-Garcia L, Chen XM, Matijevic-Aleksic N, Du M, Wu KK (1999). Suppression of inducible cyclooxygenase 2 gene transcription by aspirin and sodium salicylate. Proceedings of the national academy of sciences of the United States of America.

[9] AE AS (2015). The chemical constituents and pharmacological importance of celosia cristata–a review. The botulinum journal.

[10] AE A-S (2016). The pharmacological activities of Cuminum cyminum- A review. IOSR Journal of pharmacy.

[11] Hanif C AT, Adila S, Saeed M, Tanveer A, Ashfaq M (2012). Physico-chemical investigation and antimicrobial activity of essential oil of Cuminum cyminum L. World applied sciences journal.

[12] Geraldes AN dSA, Leal J, Mayeli Estrada-Villegas G, Lincopan N, Katti KV, Lugão AB (2016). Green nanotechnology from plant extracts: synthesis and characterization of gold nanoparticles Advances in Nanoparticles. Advances in nanoparticles.

[13] Katti K, Chanda N, Shukla R, Zambre A, Suibramanian T, Kulkarni RR, Kannan R, Katti KV (2009). Green nanotechnology from cumin phytochemicals: generation of biocompatible gold nanoparticles. International journal of green nanotechnology. Biomedicine.

[14] Selvaraj Raja VR, Varadavenkatesan Thivaharan (2017). Green biosynthesis of silver nanoparticles using Calliandra haematocephala leaf extract, their antibacterial activity and hydrogen peroxide sensing capability. Arabian journal of cemistry.

[15] Harikumar SL, Nirmala N (2013). Nanoparticles: An overview. Journal of drug delivery and therapeutics.

[16] Bruchez M MM, Gin P, Weiss S, Alivisatos AP (1998). Semiconductor nanocrystals as fluorescent biological labels. Science.

[17] Wang S MN, Kotov NA, Chen W, Studer J (2002). Antigen/antibody immunocomplex from CdTe nanoparticle bioconjugates. Nano letters.

[18] Chan WC, Nie S (1998). Quantum dot bioconjugates for ultrasensitive nonisotopic detection. Science.

[19] Mah C ZI, Fraites TJ, Dobson J, Batich C, Byrne BJ (2000). Microsphere- mediated delivery of recombinant AAV vectors in vitro and in vivo. Molecular therapy.

[20] Panatarotto D PC, Hoebeke J, Brown F, Kramer E, Briand JP, Muller S, Prato M, Bianco A (2003). Immunization with peptide-functionalized carbon nanotubes enhance virus-specific neutralizing antibody responses. Chemistry and biology.

[21] Edelstein RL, Tamanaha CR, Sheehan PE, Miller MM, Baselt DR, Whitman LJ, Colton RJ (2000). The BARC biosensor applied to the detection of biological warfare agents. Biosens bioelectron.

[22] Nam JM, Thaxton CS, Mirkin CA (2003). Nanoparticle-based bio-bar codes for the ultrasensitive detection of proteins. Science.

[23] Moldovan B DL, Vulcu A, Olenic L, Perde-Schrepler M, Fischer-Fodor E, Baldea I, Clichici S, Filip GA (2017). In vitro and in vivo anti-inflammatory properties of green synthesized silver nanoparticles using Viburnum opulus L fruits extract. Materials science and engineering. C, materials for biological applications.

[24] Khoobchandani M, Katti KK, Karikachery AR, Thipe VC, Srisrimal D, Dhurvas Mohandoss DK, Darshakumar RD, Joshi CM, Katti KV (2020). New approaches in breast cancer therapy through green nanotechnology and nano-ayurvedic medicine-pre-clinical and pilot human clinical investigations. International journal of nanomedicine.

[25] Arvizo R, Bhattacharya R, Mukherjee P (2010). Gold nanoparticles: opportunities and challenges in nanomedicine. Expert opinion on drug delivery.

[26] Singh RP GH, Mruthunjaya K (2017). Cuminum cyminum– A popular spice: An Updated Review. Pharmacognosy journal.

[27] Xie ZM, Wang XM, Xu N, Wang J, Pan W, Tang XH, Zhou ZQ, Hashimoto K, Yang JJ (2017). Alterations in the inflammatory cytokines and brain-derived neurotrophic factor contribute to depression-like phenotype after spared nerve injury: improvement by ketamine. Scientific reports.

[28] Edwards RR, Kronfli T, Haythornthwaite JA, Smith MT, McGuire L, Page GG (2008). Association of catastrophizing with interleukin-6 responses to acute pain. Pain.

[29] Chamkouri N, Naghashpour M, Adelipour M, Mohammadi A, Seyedsadjadi N, Oliveira B, Golabi S (2021). Cuminum cyminum L. -mediated synthesis of silver nanoparticles: Their characterization and effect on formalin-induced nociceptive response in male rats. Biological trace element research.

[30] Wu PC, Shieh DB, Hsiao HT, Wang JC, Lin YC, Liu YC (2018). Magnetic field distribution modulation of intrathecal delivered ketorolac iron-oxide nanoparticle conjugates produce excellent analgesia for chronic inflammatory pain. Journal of nanobiotechnology.

[31] Bahamonde J, Brenseke B, Chan MY, Kent RD, Vikesland PJ, Prater MR (2018). Gold nanoparticle toxicity in mice and rats: species differences. Toxicologic pathology.

[32] Golabi S, Hasanpour Ezati M, Azhdari H, Rohampour K, Radjabian T, Ekhteraie Tousi S (2010). Anti-nociceptive activity of regenerated Drosera spatulata aqueous extract by rat formalin test. Journal medicinal plants.

[33] Shariat HS (1992). Qualitative and quantitative evaluation of the active constituents and control methods for medicinal plants. Isfahan: Mani Publication.

[34] Golabi S MA, Chamkouri N (2019). Investigation of anti-inflammatory effect of aqueous extract of Cuminum cyminum L by formalin inflammatory model in male rats. Journal of Medicinal Plants.

[35] Al-Shawi SG, Al-Younis ZK, Al-Kareem NF (2017). Study of cumin antibacterial and antioxidant activity of alcoholic and aqueous extracts. Pakistan journal of biotechnology.

[36] Sneha K, Sathishkumar M, Lee SY, Bae MA, Yun YS (2011). Biosynthesis of Au nanoparticles using cumin seed powder extract. Journal of nanoscience and nanotechnology.

[37] Pandey S, Patel MK, Mishra A, Jha B (2015). Physio-Biochemical composition and untargeted metabolomics of Cumin (Cuminum cyminum ) make it promising functional food and help in mitigating salinity stress. PloS one.

[38] Miguel MG (2010). Antioxidant and anti-inflammatory activities of essential oils: a short review. Molecules.

[39] Ahn S, Singh P, Castro-Aceituno V, Yesmin Simu S, Kim YJ, Mathiyalagan R, Yang DC (2017). Gold nanoparticles synthesized using Panax ginseng leaves suppress inflammatory-mediators production via blockade of NF-kappaB activation in macrophages. Artificial cells, nanomedicine, and biotechnology.

[40] Naz F, Koul V, Srivastava A, Gupta YK, Dinda AK (2016). Biokinetics of ultrafine gold nanoparticles (AuNPs) relating to redistribution and urinary excretion: a long-term in vivo study. Journal of drug targeting.

[41] Agarwal H, Nakara A, Shanmugam VK (2019). Anti-inflammatory mechanism of various metal and metal oxide nanoparticles synthesized using plant extracts: A review. Biomedicine and pharmacotherapy.

[42] Taherian AA EH, Sadeghi H (2007). Assessment of Aqueous Extract of seed of Cuminum cyminum L on neurogenic and inflammatory pain in mice. Journal of medicinal plants.

[43] Verma A, Uzun O, Hu Y, Hu Y, Han HS, Watson N, Chen S, Irvine DJ, Stellacci F (2008). Surface-structure-regulated cell-membrane penetration by monolayer-protected nanoparticles. Nature materials.

[44] Chou LY, Ming K, Chan WC (2011). Strategies for the intracellular delivery of nanoparticles. Chemical society reviews.

[45] Nirmala MJ, Durai L, Rao KA, Nagarajan R (2020). Ultrasonic nanoemulsification of Cuminum cyminum essential oil and its applications in medicine. International journal of nanomedicine.

[46] Thipe VC, Panjtan Amiri K, Bloebaum P, Raphael Karikachery A, Khoobchandani M, Katti KK, Jurisson SS, Katti KV (2019). Development of resveratrol-conjugated gold nanoparticles: interrelationship of increased resveratrol corona on anti-tumor efficacy against breast, pancreatic and prostate cancers. International journal of nanomedicine.

[47] Zhang L, Radovic-Moreno AF, Alexis F, Gu FX, Basto PA, Bagalkot V, Jon S, Langer RS, Farokhzad OC (2007). Co-delivery of hydrophobic and hydrophilic drugs from nanoparticle-aptamer bioconjugates. ChemMedChem.

[48] Feghali CA, Wright TM (1997). Cytokines in acute and chronic inflammation. Frontiers in bioscience.

[49] Yasaman Husseini HS (2015). Analgesic and anti-inflammatory activities of hydro-alcoholic extract of Lavandula officinalis in mice: possible involvement of the cyclooxygenase type 1 and 2 enzymes. Brazilian journal of pharmacognosy.

[50] Al-Snafi AE (2018). Arabian medicinal plants with antiinflammatory effects- plant based review (part 1). IOSR journal of pharmacy.

[51] Bhat SP RW, Kumar Anil (2014). Efect of cuminum cyminum L Seed extracts on pain and inﬂammation. Journal of natural remedies.

[52] Sayah M PA, Kamalinezhad M (2002). Anti-nociceptive effect of the fruit essential oil of cuminum cyminum L in rat. Iranian biomedical journal.

[53] Gias ZT, Afsana F, Debnath P, Alam MS, Ena TN, Hossain MH, Jain P, Reza HM (2020). A mechanistic approach to HPLC analysis, antinociceptive, anti-inflammatory and postoperative analgesic activities of panch phoron in mice. BMC complementary medicine and therapies.

[54] Doak GJ, Sawynok J (1997). Formalin-induced nociceptive behavior and edema: involvement of multiple peripheral 5-hydroxytryptamine receptor subtypes. Neuroscience.

[55] Shibata M, Ohkubo T, Takahashi H, Inoki R (1989). Modified formalin test: characteristic biphasic pain response. Pain.

[56] Chapman V, Dickenson AH (1992). The spinal and peripheral roles of bradykinin and prostaglandins in nociceptive processing in the rat. European journal of pharmacology.

[57] Malmberg AB, Yaksh TL (1995). Cyclooxygenase inhibition and the spinal release of prostaglandin E2 and amino acids evoked by paw formalin injection: a microdialysis study in unanesthetized rats. The journal of neuroscience.

[58] Srivastava KC (1989). Extracts from two frequently consumed spices--cumin (Cuminum cyminum) and turmeric (Curcuma longa)-inhibit platelet aggregation and alter eicosanoid biosynthesis in human blood platelets. Prostaglandins, leukotrienes, and essential fatty acids.

